# Learning lessons from a toddler

**DOI:** 10.7554/eLife.59587

**Published:** 2020-06-10

**Authors:** Madhumala K Sadanandappa

**Affiliations:** Department of Molecular and Systems Biology, Geisel School of Medicine at DartmouthHanoverUnited States

**Keywords:** parent and scientist, inclusion, diversity, motherhood, international move

## Abstract

Struggling to get her research project up and running in a new country, a mother gets inspiration from her young daughter.

A few years ago, armed with a fellowship from the Human Frontier Science Program, I decided to leave India, my home country, and move to an Ivy League institution in the United States as a postdoctoral research fellow. Everything was perfect – or seemed to be. Confident in my research and academic skills, I was looking forward to doing good science and achieving my career goals. My beloved partner and young daughter, who stood by me through good and bad times, were also moving with me.

**Figure 1:**
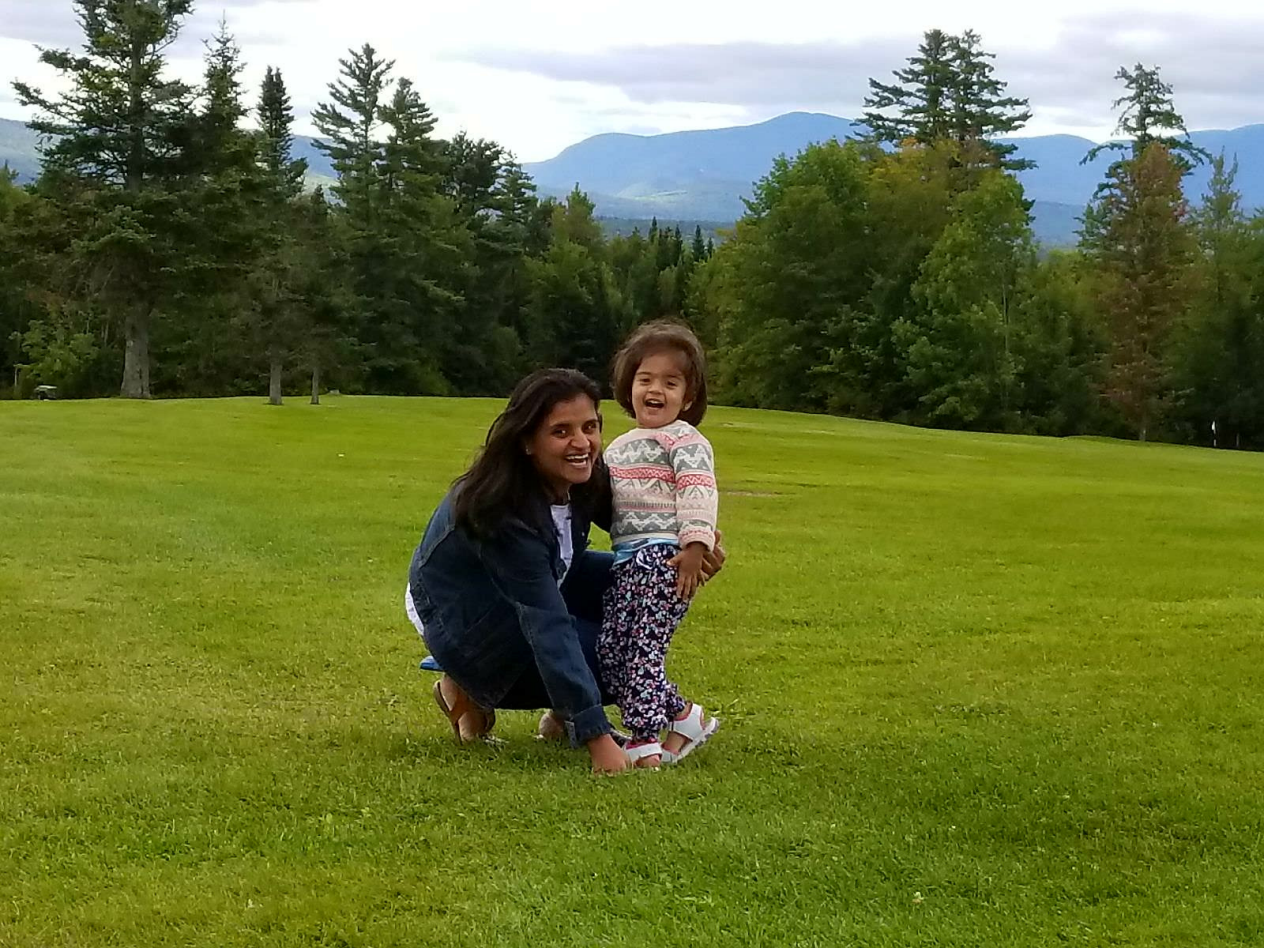
Madhu and her daughter.

Image credit: Shivaprasad H Sathyanarayana

Thousands of miles away from home, trying to settle in our new lives, our toddler started to throw tantrums. The unfamiliar language and weather, the new food and faces, and the absence of friends and family were all overwhelming for a young child. As an inexperienced parent, the next few months were both miserable and unforgettable. Fortunately, this phase did not last long: nurtured by a loving daycare, quickly learning the language and making new friends, my daughter moved on so fast. Her mother, however, did not.

More than the failed experiments or unexpected work problems, I was broken by the system of a host country that sadly considered outsiders as aliens.

I was aimlessly staring at the leafless winter trees one day, when my daughter asked: “Mamma, are you OK?”. “Yes, I am fine!” I replied, failing to convince her. She cracked a joke to lift my mood, and reassured by my smile, disappeared to play with her friends. A few minutes later, still frozen in place, I reflected on our conversation; my thoughts, much like the winter skies, began to clear. Watching the kids enjoy the snow, I realized that while my toddler was not struggling anymore, I was.

More than the failed experiments or unexpected work problems, I was broken by the system of a host country that sadly considered outsiders as aliens. I had worked abroad before, in Germany and Ireland, and my positive experiences there in terms of collaborative and inclusive working environment made me overlook, perhaps, that things could be any different. I had prepared for the scientific problem I was planning to address, but not for the new system in which I would be working.

In the end, it does not matter whether I had failed to adapt to the new culture, or whether the system had failed to create inclusiveness – maybe it was both. The outcome was that my professional and personal life begun to fall apart in a new system, a new institution, and a new lab. My motivation stagnated: I was failing to cope with academic failure, and found myself withdrawn and isolated, lacking the support of trustworthy peers and colleagues. My energy levels dropped, I was depressed and stopped sleeping properly. Not surprisingly, I began to see myself as a failure, incapable of tackling the challenges presented to me by the system. I needed help, yet I was uncomfortable looking for it, worrying that openly discussing my struggles at work would damage my career.

To avoid working with difficult people, and to escape conflicts and responsibilities, I started to spend more time with my toddler. Simply being present was enough for her to tell me stories and teach me fun things, from making slime to playing with dollies. And as we were sharing more and more time quality time together, she helped me remember the child I used to be. I was a little girl with big dreams, raised in a society where girls were prepared mainly for home making and often discouraged from attending colleges. In those testing times, my father broke cultural norms: he supported my education, nurtured my curiosity, built my confidence and encouraged my individuality. My daughter reminds me of that curiosity-driven young girl, who tackled challenges with persistent effort and hard work. She helped me take a step back and reflect on my passion, struggles, potential, accomplishments, and values. Re-learned from a toddler, the forgotten lesson “expect the unexpected, accept it, and move on”, powered me – a well-educated, graduate mother – through uncharted waters.

The path to recovery was neither linear nor easy, encompassing several months of emotional roller coasters. But with the help of my supportive advisor, I changed my research direction, and I invested my time and energy in building reliable inter-personal relationships, in and out of the work place. Opportunities opened up after I started to volunteer for a professional organization, and I found a new sense of purpose and satisfaction mentoring youth from disadvantaged backgrounds. Slowly, I began to see the light at the end of the tunnel, overcoming my setbacks and insecurities. It was a long way to go, but I am not a failure – least of all in the eyes of my daughter, who is always so proud of me.

